# Selective Arterial Embolization for Persistent Urinoma Following Traumatic Renal Injury: A Case Report and Review of the Literature

**DOI:** 10.7759/cureus.87525

**Published:** 2025-07-08

**Authors:** Ghadir H Badr, Areej M Alamri, Yousef A Ekhmimi, Lojain T Alharbi, Nawaf AlShahwan

**Affiliations:** 1 General Surgery, Ministry of Health, King Fahad Hospital, Medina, SAU; 2 Radiology, Ministry of Health, King Fahad Hospital, Medina, SAU; 3 Surgery, College of Medicine, King Khalid University Hospital, Riyadh, SAU

**Keywords:** nephrectomy, renal trauma, retained renal remnant, selective arterial embolization (sae), urinoma

## Abstract

Traumatic and iatrogenic renal injuries, particularly high-grade trauma and complications following nephron-sparing surgeries, pose significant clinical challenges. Complications such as urinomas, pseudoaneurysms, and arteriovenous fistulas (AVFs) can lead to serious morbidity if not promptly addressed. Selective arterial embolization (SAE) has emerged as a minimally invasive alternative to surgery, offering effective hemorrhage control while preserving renal function. We report the case of a 51-year-old male patient who sustained a Grade V shattered left kidney following blunt trauma. The patient underwent emergency laparotomy with left nephrectomy. Postoperatively, a persistent urinoma developed from a retained kidney remnant. SAE was successfully performed to manage this complication, achieving complete resolution without further surgical intervention. SAE demonstrates high technical success and clinical efficacy in treating renal vascular complications after trauma or surgery. It offers the advantages of nephron preservation, reduced morbidity, and adaptability through various embolic agents. Despite risks such as post-embolization syndrome and potential non-target embolization, SAE remains the preferred approach in hemodynamically stable patients or those at high risk for reoperation. Early diagnosis and timely intervention are essential for optimal outcomes. SAE is a safe, effective, and nephron-sparing treatment modality for complex renal vascular injuries and complications. It should be considered a first-line therapeutic option in appropriate clinical settings, especially when surgical reintervention poses high risks.

## Introduction

Traumatic and iatrogenic renal injuries present complex clinical challenges, particularly in hemodynamically unstable patients or those experiencing postoperative complications. High-grade renal trauma and nephron-sparing surgical procedures, such as partial nephrectomy, can lead to serious sequelae, including urinomas, vascular pseudoaneurysms, and arteriovenous fistulas (AVFs) [1-3]. Accurate grading and timely management of these injuries are critical, as outlined by Buckley and McAninch and Chien et al., who emphasize the importance of tailored interventions based on injury severity [4,5]. If not promptly and appropriately managed, these complications may result in persistent hemorrhage, infection, or irreversible renal damage [6,7]. Traditionally, these conditions were managed surgically; however, advances in interventional radiology have shifted treatment paradigms toward minimally invasive strategies. Among these, selective arterial embolization (SAE) has emerged as a valuable technique, providing effective hemorrhage control while preserving renal parenchyma [2,8-10].

SAE has demonstrated clinical success in various contexts, from managing post-traumatic pseudoaneurysms to treating iatrogenic vascular injuries following nephrectomy [3,6,9]. Its role is particularly important when surgical reintervention carries high risk due to prior operative complexity or patient instability [2,7,10]. Herein, we explore the application of arterial embolization for treating a persistent urinoma arising from a retained kidney remnant in a patient with severe blunt renal trauma who underwent subtotal nephrectomy.

## Case presentation

A 51-year-old male patient presented to the emergency department after falling from a camel, complaining of severe abdominal pain. On arrival, he was alert with a Glasgow Coma Scale (GCS) score of 15/15. Vital signs revealed a blood pressure of 118/71 mmHg, heart rate of 108 beats per minute, oxygen saturation of 96% on room air, and respiratory rate of 21 breaths per minute. Physical examination demonstrated diffuse abdominal tenderness with guarding but no external signs of trauma. A Focused Assessment with Sonography for Trauma (FAST) revealed free fluid in the splenorenal recess. Chest and pelvic radiographs were unremarkable.

**Figure 1:**
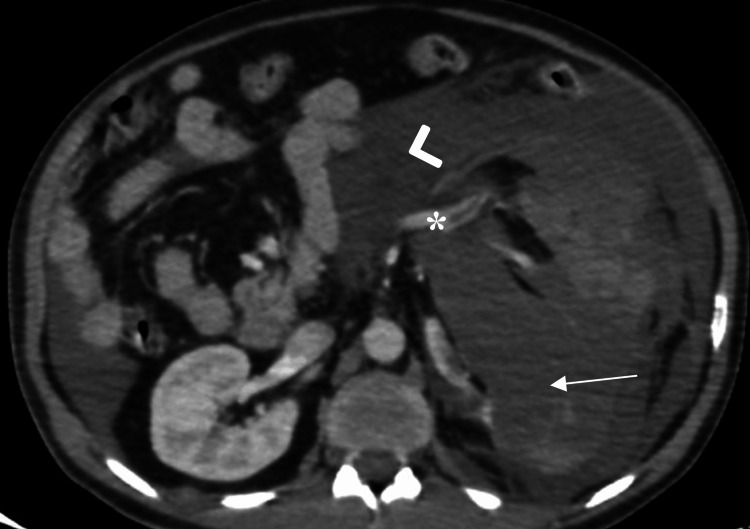
Axial contrast-enhanced computed tomography (CT) scan of the abdomen shows a shattered left kidney (arrow), with a large perinephric hematoma/urinoma (arrowhead), filling defect in the left renal vein indicative of vascular injury/thrombus (asterisk).

The patient initially responded to transfusion with two units of packed red blood cells (PRBCs), stabilizing his condition sufficiently to undergo a whole-body computed tomography (CT) scan. A non-contrast CT brain was unremarkable. Contrast-enhanced CT of the abdomen and pelvis revealed a Grade V shattered left kidney with abdominopelvic fluid collections suggestive of hematoma/urinoma. Other intra-abdominal organs were unremarkable (Figure 1).

Despite ongoing resuscitation, the patient’s condition deteriorated, with hypotension and worsening venous blood gas (VBG) parameters. An emergency exploratory laparotomy was indicated, and a massive transfusion protocol was activated. Under general anesthesia, a midline laparotomy was performed. An expanding retroperitoneal hematoma was identified on the left side. A medial visceral rotation was executed to expose the left retroperitoneum. Temporary hemostasis was achieved with packing.

**Figure 2:**
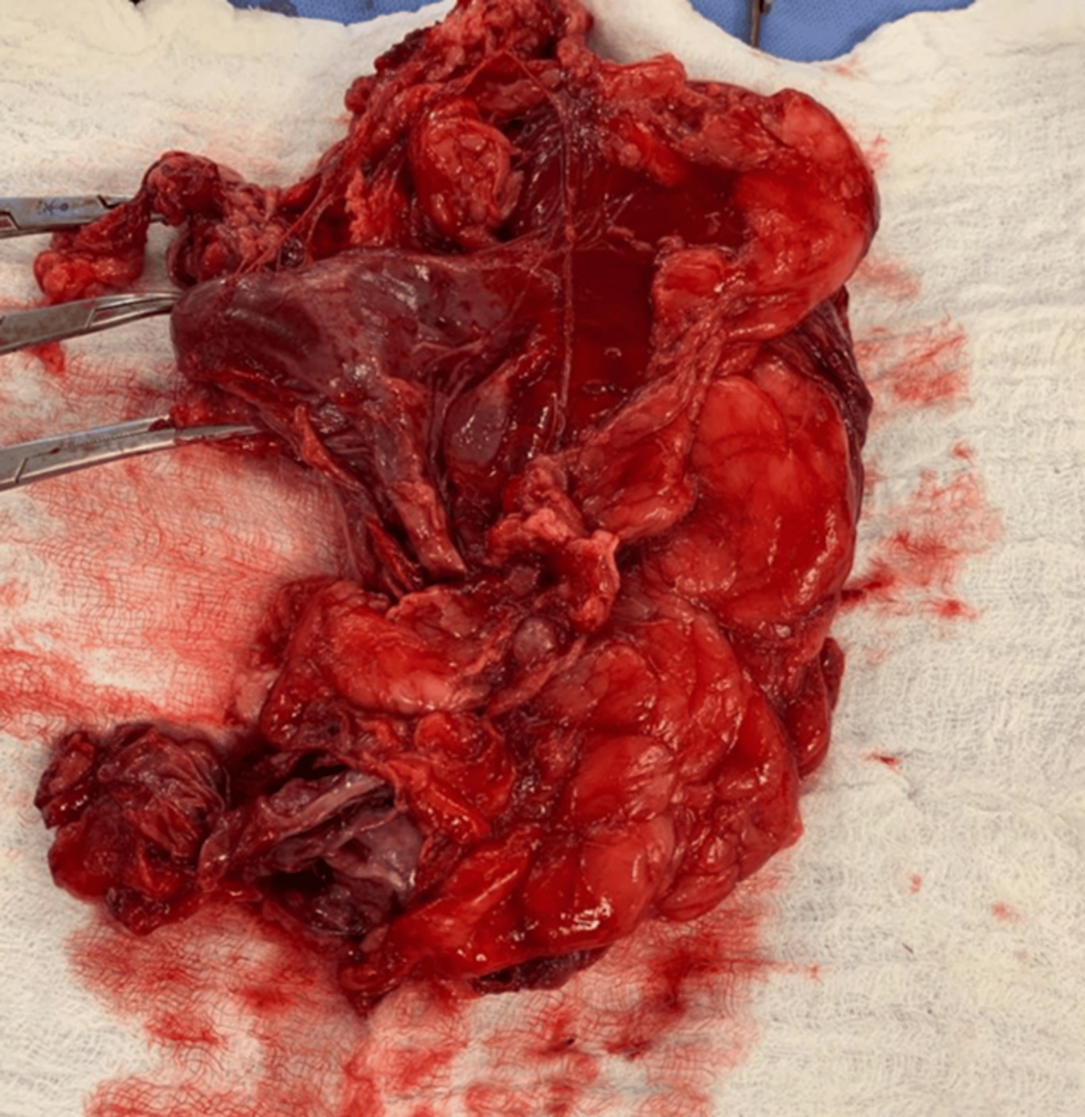
Left kidney, post-nephrectomy

The left kidney was found to be shattered and non-viable, with active arterial bleeding. The left renal artery was dissected, ligated with 2-0 silk, and divided. The left renal vein and ureter were similarly identified, ligated, and divided. A total left nephrectomy was completed (Figure 2).

Hemostasis was confirmed, including inspection of the splenic hilum and hepatic dome. No other intra-abdominal injuries were found. A drain was placed near the nephrectomy bed. The abdomen was irrigated, and the fascia was closed with polydioxanone (PDS) sutures; skin closure was performed using staples.

Intraoperatively, the patient received 10 units of PRBCs, platelets, and fresh frozen plasma (FFP). He remained hemodynamically stable and was transferred to the intensive care unit (ICU) postoperatively.

On postoperative day (POD) 1, the patient was extubated and was alert, oriented, and vitally stable with GCS 15/15. On POD 2, he tolerated oral intake without vomiting. The abdominal dressing was removed, revealing a clean and dry wound, and laboratory investigations were acceptable. He was subsequently transferred to the general ward.

By POD 5, the patient was tolerating oral intake, ambulating, and passing bowel movements. However, persistently high output from the abdominal drain warranted further investigation. Analysis of the drain fluid revealed an elevated drain-to-serum creatinine ratio (drain fluid creatinine: 5.6 mg/dL; serum creatinine: 0.7 mg/dL), suggestive of a urinary leak.

**Figure 3:**
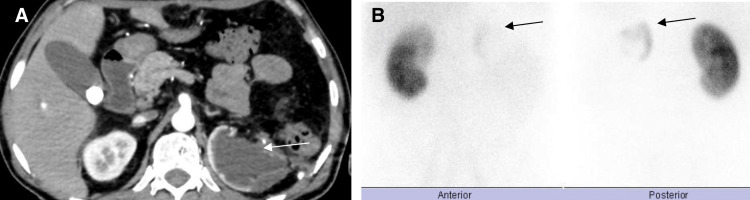
(A) Axial contrast-enhanced CT scan of the abdomen shows remnant left kidney tissue (arrow). (B) DMSA renal scan demonstrates radiotracer uptake at the left upper renal region (arrows). CT: computed tomography; DMSA: dimercaptosuccinic acid

CT abdomen and pelvis was done and showed left kidney upper pole remnant tissue, which was confirmed later by dimercaptosuccinic acid (DMSA) renal scan (Figure 3).

**Figure 4:**
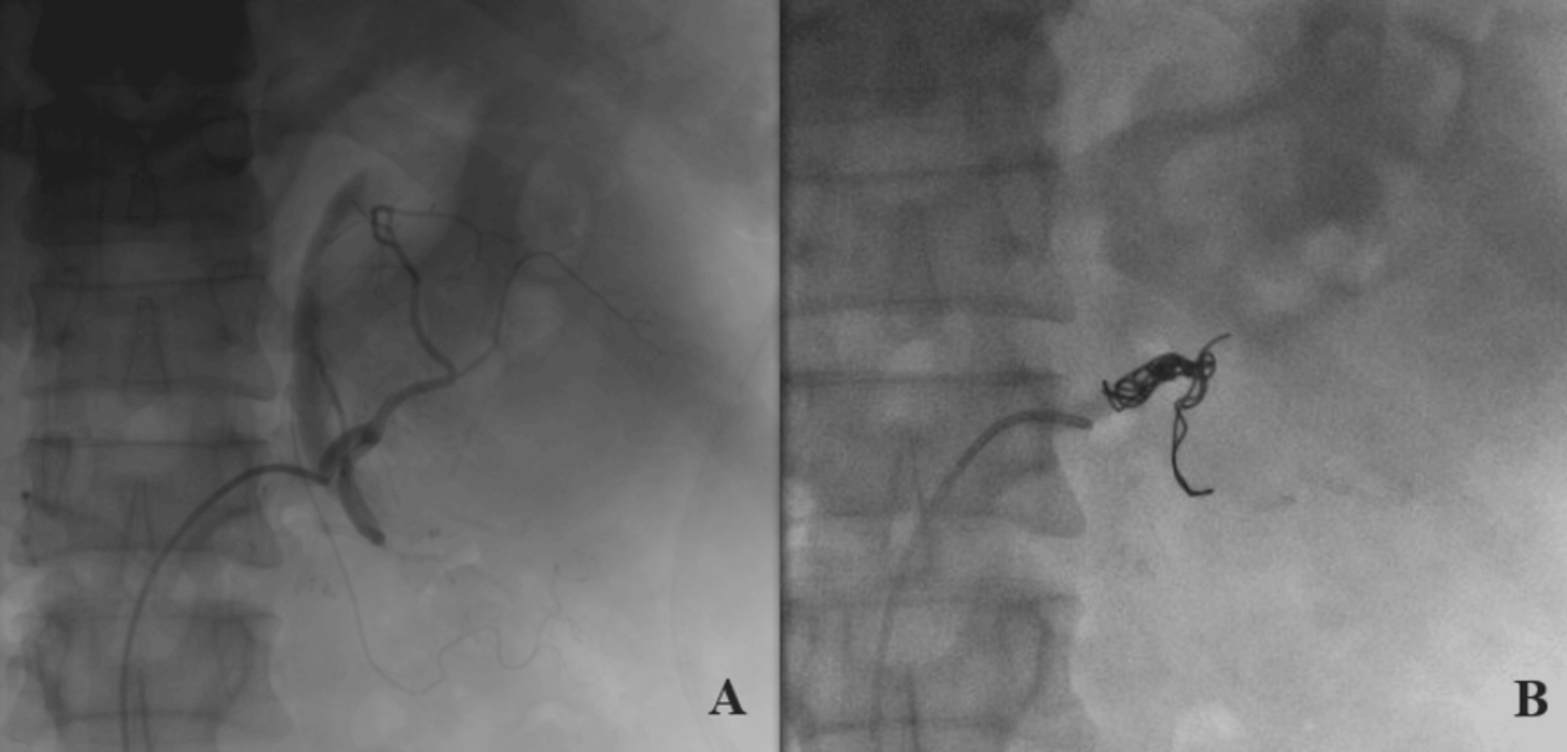
(A) Selective angioembolization of the left renal artery with residual renal parenchyma. (B) Successful embolization of the left renal artery with microcoils.

On POD 11, due to persistent high-output drainage, the patient underwent successful angioembolization of the remnant left renal tissue using coils and Gelfoam, performed by interventional radiology (Figure 4). The procedure was uneventful and effectively occluded the remnant tissue.

Post-embolization, the patient remained stable without complications. Drain output decreased significantly to 20 mL/day and was subsequently removed on POD 12. The patient was discharged in good condition and scheduled for outpatient follow-up.

At the two-week follow-up visit, the patient reported no new complaints or urinary symptoms. Clinical examination revealed a soft, non-tender abdomen. Follow-up ultrasonography showed minimal residual fluid collection.

## Discussion

Renal trauma, particularly when compounded by vascular or urinary tract injuries, continues to pose significant challenges in both trauma and surgical practice. These injuries range from minor contusions and lacerations to complex disruptions of renal parenchyma, vasculature, and collecting systems. The American Association for the Surgery of Trauma (AAST) classified renal injury into five grades [11].

While low-grade injuries, classified as Grades I and II, can often be managed conservatively with observation, bed rest, and supportive care, higher-grade injuries (Grades IV and V), especially those complicated by ongoing hemorrhage or urinary extravasation, frequently necessitate timely and targeted interventions [1,5,6,8].

High-grade renal trauma can result in complications such as urinomas, pseudoaneurysms, and AVFs. These sequelae may compromise renal perfusion, precipitate significant blood loss, and lead to infection, loss of renal function, or even death if not appropriately managed. Historically, the standard treatment for such complex injuries involved operative exploration and nephrectomy, which, while often effective, carries a high risk of morbidity, longer hospital stays, greater blood loss, and the permanent loss of nephron mass [6,7]. Additionally, surgical management may not always be ideal in hemodynamically unstable or critically ill patients, or those with single kidneys or pre-existing renal impairment.

In recent decades, the field of interventional radiology has introduced minimally invasive alternatives that offer effective and targeted treatment with fewer complications. Among these, SAE has emerged as a cornerstone technique in the non-operative management of renal vascular injuries, both traumatic and iatrogenic [2,6,9]. SAE is a catheter-based procedure that involves the selective occlusion of bleeding arteries or damaged vascular segments using embolic agents. This approach enables rapid control of hemorrhage while preserving as much functional renal tissue as possible.

Numerous studies have demonstrated the effectiveness of SAE in managing vascular complications following renal trauma or surgery. Pozzi et al. reported success in using SAE to treat bleeding from retained renal remnants following trauma or subtotal nephrectomy, thereby avoiding reoperation in critically ill patients [1]. Similarly, Tinto et al. and Zhu et al. documented SAE’s efficacy in treating postoperative pseudoaneurysms and AVFs, with excellent outcomes and minimal complication rates [12,13].

Loffroy et al., in a large retrospective analysis, found that SAE achieved technical success in 96% of patients after the initial procedure, with repeat embolization increasing this rate to 100% in patients with persistent bleeding or complex vascular lesions [2]. This high success rate has made SAE a preferred modality, even in emergent situations. Ghoneim et al. further supported these findings by showing rapid hemostasis and functional preservation of renal parenchyma in patients who underwent embolization for postoperative bleeding [6]. In a comprehensive systematic review, Jain et al. affirmed SAE as the treatment of choice in nearly all cases of pseudoaneurysms following partial nephrectomy, given its safety profile and clinical effectiveness [9].

One of the most notable strengths of SAE is its adaptability to the patient's anatomy and clinical scenario. A wide variety of embolic materials can be employed-including metallic coils, polyvinyl alcohol particles (embospheres), gelatin sponges, and liquid agents like n-butyl cyanoacrylate (NBCA) and Onyx^®^ (Medtronic, Minneapolis, MN, USA). Each agent has distinct properties, allowing interventional radiologists to tailor the approach based on lesion size, vascular flow dynamics, and the proximity of vital structures. Coils are the most frequently used embolic due to their precise deployment and ability to occlude targeted vessels, particularly pseudoaneurysms [6,9]. Liquid embolics offer deeper penetration into smaller, high-flow AVFs, but their use requires considerable expertise to avoid inadvertent non-target embolization. According to Loffroy et al., there is no significant difference in outcomes based on the embolic material used, which highlights the importance of operator experience and lesion-specific planning over material choice alone [2].

Beyond its immediate technical efficacy, SAE offers substantial benefits in preserving renal function, making it especially valuable in patients with solitary kidneys, pre-existing chronic kidney disease, or bilateral injuries. Studies by Gahan et al. and Hyams et al. found no significant decline in glomerular filtration rate (GFR) or serum creatinine levels following embolization, even in cases involving large volumes of embolized tissue. Loffroy et al. further confirmed that although embolization led to an average 13.8% reduction in renal volume, there was no significant compromise in creatinine clearance during medium-term follow-up [2,7,8]. Similarly, a prospective study by Sauk and Zuckerman demonstrated minimal functional impairment and low complication rates in patients who underwent renal artery embolization for various indications, underscoring SAE’s nephron-sparing nature [14].

Despite its many advantages, SAE is not without risks. The most common complication is post-embolization syndrome, which includes symptoms such as fever, flank pain, nausea, and leukocytosis. This self-limiting condition typically resolves within a few days with conservative management. More serious but rare complications include infarction of healthy renal tissue, non-target embolization of nearby organs, renal abscess formation, persistent bleeding due to incomplete embolization, and contrast-induced nephropathy [2,8]. These risks emphasize the need for skilled operators, meticulous technique, and thorough pre-procedural planning using high-quality imaging. Furthermore, careful attention must be paid to patients with pre-existing renal dysfunction or diabetes, as they are at greater risk of nephrotoxicity from iodinated contrast agents used during angiography and CT imaging [3,7].

Timely diagnosis and intervention are essential for optimal outcomes. Vascular complications such as pseudoaneurysms and AVFs may present several days to weeks following the initial trauma or surgery, often with non-specific symptoms such as flank pain, hematuria, or persistent drain output. High clinical suspicion, prompt imaging (such as contrast-enhanced CT or diagnostic angiography), and early interventional management are crucial to prevent delayed rupture or ongoing blood loss. Even asymptomatic lesions, if identified on routine follow-up imaging, may warrant prophylactic embolization to mitigate future risks [2,6,9].

Emerging technologies such as contrast-enhanced ultrasound (CEUS) have shown promise as non-nephrotoxic alternatives for evaluating renal vascular lesions. CEUS offers excellent real-time resolution and can effectively detect pseudoaneurysms, especially in patients with contraindications to contrast-enhanced CT or MRI. However, its use remains limited due to operator dependence and restricted availability in many institutions [8,9,15,16].

## Conclusions

SAE represents a safe, effective, and minimally invasive modality for managing complex renal vascular complications arising from trauma or iatrogenic injury. Its ability to achieve rapid hemostasis while preserving renal parenchyma makes it especially valuable in patients who are poor surgical candidates or have undergone multiple abdominal interventions. With high technical success rates and minimal impact on long-term renal function, SAE has become a cornerstone in contemporary renal trauma and postoperative care. Nevertheless, careful patient selection, timely diagnosis, and operator expertise remain critical to optimize outcomes and minimize complications. Future prospective studies are warranted to further refine embolization techniques and develop standardized treatment protocols to enhance patient care.
